# Ubiquitin domain proteins in disease

**DOI:** 10.1186/1471-2091-8-S1-S1

**Published:** 2007-11-22

**Authors:** Louise Madsen, Andrea Schulze, Michael Seeger, Rasmus Hartmann-Petersen

**Affiliations:** 1 Insitute of Molecular Biology and Physiology, University of Copenhagen, Universitetsparken 13, DK-2100 Copenhagen, Denmark; 2 Charité – Universitätsmedizin Berlin, Campus Charité Mitte, Institut für Biochemie, Monbijoustr. 2, 10117 Berlin, Germany

## Abstract

The human genome encodes several ubiquitin-like (UBL) domain proteins (UDPs). Members of this protein family are involved in a variety of cellular functions and many are connected to the ubiquitin proteasome system, an essential pathway for protein degradation in eukaryotic cells. Despite their structural similarity, the UBL domains appear to have a range of different targets, resulting in a considerable diversity with respect to UDP function. Here, we give a short summary of the biochemical and physiological roles of the UDPs, which have been linked to human diseases including neurodegeneration and cancer.

**Publication history:** Republished from Current BioData's Targeted Proteins database (TPdb; ).

## Introduction

Ubiquitin is a small and phylogenetically conserved eukaryotic protein known to covalently modify proteins and thereby mark them for destruction by the 26S proteasome [1]. This process, termed ubiquitylation, is also involved in the regulation of many other cellular processes including endocytosis and transcription [1].

Ubiquitin ligation is accomplished in multiple steps [2], the first of which involves ATP-dependent activation of ubiquitin by a ubiquitin activating enzyme (E1). Second, the activated ubiquitin is transferred to a ubiquitin conjugating enzyme (E2). A ubiquitin protein ligase (E3) then associates with both the ubiquitin-charged E2 and the substrate, facilitating the formation of an isopeptide bond between the C-terminus of ubiquitin and an amino group (typically a lysine residue) either directly on the target protein or on the last ubiquitin moiety of an attached polyubiquitin chain [2]. Several such rounds of conjugation yield substrates carrying chains of ubiquitin moieties, which may be elongated further by the action of a ubiquitin chain elongation factor (E4) [3]. Combinations of E2 and E3 enzymes (of which there are hundreds encoded in the human genome) primarily provide the substrate specificity of the ubiquitylation system [2].

The formation of polyubiquitin chains is a reversible process and several deubiquitylating enzymes (DUBs) play important roles in trimming the chains on target proteins [4].

Different ubiquitin lysine residues are used in the formation of polyubiquitin chains, with various outcomes. For example, whereas Lys48-linked polyubiquitin chains target proteins for degradation by the 2.5 MDa 26S proteasome in an ATP-dependent manner [5], linking through Lys63 plays a role in NFκB-signalling and the formation of Lewy bodies in patients with Parkinson's disease [6-8]. Modification of proteins with a single ubiquitin moiety (monoubiquitylation) also plays an important role, namely in endocytosis and transcription [9].

Ubiquitin is a stable and compact protein consisting of two α-helices and five β-strands arranged in the order ββαββαβ to form the ubiquitin superfold [10]. Recently, several ubiquitin-like proteins have been identified, which despite a relatively low sequence similarity, all display the ubiquitin superfold [11]. These proteins are generally divided into two groups: ubiquitin-like modifiers (UBLs) and ubiquitin-like domain proteins (UDPs) [12]. In addition to ubiquitin itself, the group of UBLs can become covalently attached to substrate proteins in a similar manner to ubiquitylation, and includes proteins such as NEDD8, SUMO, FAT10 and others. Despite their similarities, the assorted UBLs are implicated in different cellular functions [12]. However, we shall not discuss them further here, but instead focus on the group of UDPs.

The UDPs are responsible for recruitment of ubiquitylated substrates to the proteasome [13] and bind to the 26S proteasome in a UBL-dependent manner [14]. Hence, it was thought that the UBL domain is a general proteasome binding domain. However, recently it was demonstrated that some UDPs do not interact with the proteasome [15]. Thus, despite their structural similarities to each other and ubiquitin, UDPs display striking differences on the functional level. Here we give a brief overview of a selection of UDPs involved in human diseases.

## UBL/UBA domain proteins

**Figure 1:**
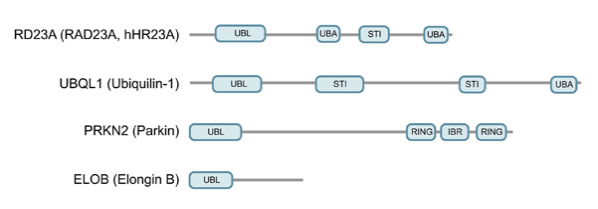
**Domain organization of selected UBL domain proteins**. The figure depicts schematic representations of the domain architecture of selected human UBL domain proteins. The domains are: UBL, ubiquitin-like; UBA, ubiquitin-associated; STI, Sti1-like; RING, ring finger; IBR, in-between ring fingers.

UDPs that contain one or more ubiquitin binding UBA (ubiquitin-associated) domain (figure 1) in addition to a UBL domain are called UBL/UBA domain proteins. The yeast proteins Rad23 and Dsk2 are the best characterized members of this group.

Precipitation experiments have shown that the UBL domain of both Rad23 and its human homologue (HHR23) interacts directly with the 26S proteasome *in vitro*[14], while the UBA domain interacts with ubiquitin [13]. UBL/UBA domain proteins were therefore proposed to function as substrate shuttles, transporting ubiquitylated proteins in the cytosol from E3 enzymes to the 26S proteasome [16]. This shuttle model was recently confirmed *in vitro* by biochemical experiments, which showed that UBL/UBA proteins are essential for degradation of a proteasome substrate [17]. Since most proteasome substrates, including cell cycle regulators, oncogenes and tumour suppressor proteins [1], are probably directed to the proteasome via the UBL/UBA proteins, drugs targeting these proteins may prove useful in treating cancer.

Despite the fact that none of the UBL/UBA proteins are essential in yeast, combined loss of Rad23, Dsk2 and the proteasome's ubiquitin receptor Rpn10/S5a results in mitotic arrest [13]. This indicates that though UBL/UBA proteins are redundant and have overlapping substrate specificity, some seem to be relatively specific for certain target proteins. This has already been shown by pulse-chase experiments for certain cell cycle-related proteins, such as Sic1 and Cln2 [17]. Therefore, specific rather than general targeting of UBL/UBA proteins could be a more effective approach in cancer therapy.

In precipitation experiments the UDPs Rad23, Dsk2 and their human orthologues HHR23 and ubiquilin-1 interact with the 26S proteasome in a UBL domain-dependent manner [13,18,19]. However, within the 26S proteasome, the UDPs appear to have distinct binding preferences. For example, yeast Rad23 and Dsk2 interact with the proteasome subunit Rpn1/S2 [20,21], whereas human HHR23 interacts with the second ubiquitin interaction motif (UIM) of human Rpn10/S5a [18], which is not present in yeast Rpn10/S5a [21].

Both Ufd2 (an E4) and the 26S proteasome associate with the Rad23 UBL domain in a mutually exclusive manner *in vitro*[22]. One might therefore speculate that Rad23 associates with Ufd2, possibly inhibiting its E4 activity, while binding the ubiquitylated substrate via the UBA domains. Eventually, Rad23 will dissociate from Ufd2 and dock at the 26S proteasome, thus delivering the ubiquitylated cargo [23].

In addition to the 26S proteasome and ubiquitylated substrate, HHR23 also interacts with ataxin-3 *in vitro*, a protein involved in the development of the neurodegenerative Machado-Joseph disease [24]. Similar to deubiquitylating enzymes, ataxin-3 contains ubiquitin interaction motifs (UIMs) and a protease domain. Surprisingly, the HHR23 UBL domain was found to interact with the protease domain of ataxin-3 rather than with one of the UIMs [24,25]. Since ataxin-3 also associates with both the proteasome and polyubiquitin chains, it may recruit ubiquitylated substrates to the proteasome [26]. However, ataxin-3 is also a substrate of the E4 enzyme Ufd2 [27] and perhaps of HHR23 [22,23]. Therefore, further studies are necessary to understand the physiological background of the ataxin-3–HHR23 interaction.

## UBL/UBA domain proteins in disease

### Alzheimer's disease

Ubiquilins 1–4 are human homologues of yeast Dsk2. Ubiquilin-1 and ubiquilin-2 both bind the proteasome [19] and, interestingly, have both been linked to Alzheimer's disease, since they were proposed to bind presenilins and localize to Lewy bodies and neurofibrillar tangles [28]. Moreover, genetic variations in the *ubiquilin-1* gene are proposed to substantially increase the risk of developing Alzheimer's disease [29]. The generation of the C-terminus of amyloid β-protein (Aβ) is dependent on presenilins. Mutations in *presenilin-1* and *presenilin-2* lead to an increased ratio of β-APP42 to β-APP40 (also known as Aβ42 and Aβ40) from amyloid β-precursor protein [30]. Accumulation of these Aβ peptides outside the cell leads to amyloid plaques, a major lesion in Alzheimer's disease [31]. The presenilin–ubiquilin interaction was mapped to the C-terminal UBA domain of ubiquilin-1 [28]. However, in these studies, in which ubiquilin fusion proteins were used to precipitate *in vitro* translated presenilins, SDS-PAGE of the precipitates revealed a slowly migrating smear. It is therefore likely that only ubiquitylated presenilins can interact with ubiquilin-1. It is also possible that ubiquilin-1 only interacts with ubiquitylated γ-aminobutyric acid A (GABA_A_) receptors [32].

**Figure 2:**
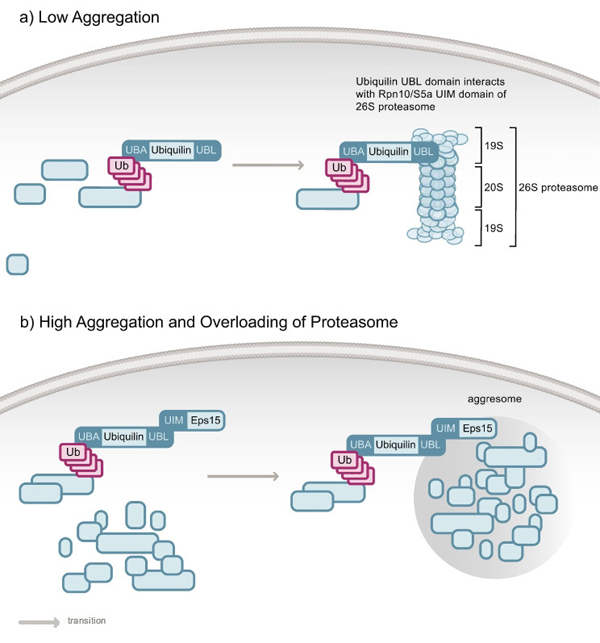
**The role of ubiquilin in protein degradation in mammalian cells**. Under normal conditions, when only low amounts of protein aggregates are present in cells (a), ubiquilin shuttles ubiquitylated proteins to the 26S proteasome via a UBL-dependent interaction with the Rpn10/S5a UIM domain. When cells contain larger amounts of aggregated protein (b), the proteasome becomes overloaded, leaving the ubiquilin UBL domain to interact with other cellular UIM proteins. These include Eps15, which instead promotes transport of aggregation-prone proteins to the aggresome. The figure is based on the model of Heir *et al.*[37].

In order to efficiently transmit synaptic signals, the amount of GABA_A_ receptor at inhibitory synapses is tightly regulated [32,33]. This is maintained by the internalization of the receptor at clathrin-coated pits [34]. Ubiquilin-1 binds GABA_A_ receptors and facilitates their membrane insertion by increasing the stability of the intracellular pool [32]. Based on these data, it is feasible that the UDP only interacts with ubiquitylated GABA_A_ receptors. As proteasomal degradation of the receptors was not observed, ubiquilin-1 may therefore also function in endocytosis [32]. Accordingly, it has been reported that ubiquilin-1 interacts with Eps15, an essential component of the clathrin-mediated endocytic pathway [35]. Eps15 contains two UIMs, and ubiquilin-1 binds to the one that is closer to the N-terminus. Besides Eps15, other UIM-containing proteins linked to the endocytic pathway also bind the UBL domain of ubiquilin-1 [35]. Although these proteins co-localize with ubiquilin-1, they were not found in endocytic compartments, but in cytoplasmic aggregates or aggresomes. Thus, ubiquilin-1 may be involved in the sequestration of certain UIM-containing endocytic proteins to ubiquitin-rich aggregates [35]. This is consistent with the finding that ubiquilin-1 and ubiquilin-2 localize to ubiquitin-positive structures and are both present in aggresomes [36]. It is possible that ubiquilin-1 targets aggregated, ubiquitylated proteins as well as UIM-containing endocytic proteins to aggresomes. This hypothesis was recently strengthened by knockdown and overexpression studies that revealed a possible dual function of the ubiquilin-1 UBL domain [37]. These studies suggest that under low levels of polyglutamine protein aggregation, ubiquilin-1 shuttles ubiquitylated proteins to the proteasome, while under higher levels of protein aggregation, the proteasome may become overloaded and alternative functions of the ubiquilin UBL domain enhanced, for example, interaction with Eps15 to promote trafficking of protein aggregates to the aggresomes (figure 2) [37]. Once the aggregated proteins reach the aggresome, their toxicity is reduced by their efficient autophagic removal [38].

### Spinocerebellar ataxia

Ubiquilin-4 is linked to spinocerebellar ataxia type 1 (SCA1) [39], an inherited neurodegenerative disease, which primarily affects the brainstem, spinocerebellar tracts and cerebellar Purkinje cells. The disease is caused by an expansion of a polyglutamine stretch within the SCA1 protein, ataxin-1. Similar to other UBL/UBA proteins, ubiquilin-4 binds the proteasome subunit Rpn10/S5a via its UBL domain and polyubiquitin chains via its UBA domain [39,40].

### Juvenile Parkinsonism

Autosomal recessive juvenile Parkinsonism (AR-JP) is an early onset and slowly progressing disease, the most common cause for which is mutations in the *PARK2* gene encoding PRKN2 (also known as parkin) [41-43]. PRKN2 contains a UBL domain and two E3-type RING-finger motifs (figure 1), and localises to the cytoplasm and centrosome/aggresome [44]. Accordingly, it was shown that PRKN2 is a functional E3 and that pathological mutations in *PARK2* impair its catalytic activity [45-47]. Several PRKN2 substrates have been identified, including the synaptic vesicle-associated GTPase CD-Crel-1, Cyclin-E, the α-synuclein-interacting protein synphilin, PaelRl and glycosylated α-synuclein [46,48-51]. Thus, loss of PRKN2 may result in the accumulation of its substrates, leading to the death of dopaminergic neurons. Supporting evidence comes from experiments in which over-expression of the PRKN2 substrate PAELR1 led to cell death in neuroblastoma cells, an effect which could be rescued by wild-type but not mutant PRKN2. [49]. Moreover, glycosylated α-synuclein was found to accumulate in the brain of PRKN2-deficient AR-JP patients [50].

Although most of the pathogenic missense mutations cluster to the RING domain-containing C-terminus of PRKN2, a few mutations have also been identified in the UBL region. NMR interaction studies have identified an interaction between the PRKN2 UBL domain and the proteasome subunit Rpn10/S5a, an observation not made with the pathogenic PRKN2 mutant R42P [52]. However, since *in vitro* precipitation assays failed to confirm the interaction [52], its significance is questionable. More recent evidence lends support to these doubts, since it was shown by immunoblotting that PRKN2 catalyses Lys63-linked ubiquitylation of synphilin-1 *in vivo*[53]. Though Lys63-linked polyubiquitin chains can support proteasomal degradation [54,55], this type of modification is also associated with endocytosis [56], indicating that perhaps PRKN2 exhibits proteasome-independent functions. Additionally, precipitation experiments demonstrated that PRKN2 binds Eps15 in a UBL domain-dependent manner [57]. Upon treating fibroblasts with epidermal growth factor (EGF), PRKN2 mediates ubiquitylation of Eps15 and thereby delays internalization of the EGF-receptor (EGFR). In PRKN2-deficient cells, however, endocytosis of EGFR is accelerated. Since EGFR stimulates neuronal survival via Akt pathway signalling, its accelerated endocytosis in mutant cells may lead to inefficient Akt signalling and reduced neuronal survival, resulting in AR-JP [57].

PRKN2 can also be destabilized by pathogenic mutations within its UBL domain [58]. However, a truncated version of PRKN2, without the UBL domain, was dramatically stabilised when compared with full-length PRKN2. The first six residues of the UBL domain were found to be responsible for destabilizing PRKN2 [59]. Thus, the PRKN2 UBL domain harbours a destabilization signal responsible for its rapid turnover. Interestingly, there is a truncated PRKN2 isoform in humans, synthesized from an internal start codon, which lacks the UBL domain. The functional significance of this PRKN2 isoform and its stability is still unclear.

### Von Hippel-Lindau disease

Von Hippel-Lindau (VHL) disease is an autosomal dominant cancer, which manifests as angiomas of the retina, hemangioblastomas of the CNS and renal clear cell carcinomas [60]. Patients with VHL disease harbour a germ line mutation in one allele of the tumour suppressor gene encoding the nuclear and cytoplasmic VHL protein [60]. VHL is the substrate binding component of the multisubunit E3 CBC^VHL^ (Cullin–Elongin-BC–VHL) [61]. In addition to VHL, the E3 complex contains the structural component Cullin-2, the catalytic subunit RBX1 (ROC1) and Elongin-B and -C [61].

CBC^VHL^ catalyses the ubiquitylation of hypoxia-inducible transcription factor α (HIFα and several other substrates [62,63]. Under normoxic conditions, HIFα is ubiquitylated by the CBC^VHL^ complex, leading to low levels of HIFα and therefore moderate expression of HIFα target genes [61]. Inactivation of VHL causes accumulation of HIFα, leading to increased transcription of HIFα target genes, most of which are angiogenic factors. This in turn causes extensive proliferation of capillaries, a crucial step in tumour development in VHL disease [61].

Elongin-B and -C are also found in other CBCs, where they either link other VHL-box proteins and Cullin-2, or SOCS-box proteins and Cullin-5 [64]. Additional functions of Elongin-B, besides its role as adapter component of CBCs, are yet to be documented, as are the functions of its UBL domain (figure 1).

## UDP disease models

Yeast has proved to be an excellent model system for the study of the UBL/UBA proteins. However, developing transgenic mouse models will further reveal important functional aspects of their molecular functions. A knockout is already available in *Rad23b* (*Mhr23b)*, the murine orthologue of *RAD23B (HHR23B*) [65], which displays impaired embryonic development and a high rate of neonatal death. In surviving animals, a variety of abnormalities are observed, including retarded growth, facial dysmorphology and male sterility [65].

To date, two *Park2* knockout mouse models have been reported [66,67], both of which are viable and fertile. The morphology and viability of dopaminergic neurons in both strains was also unaltered, even in aged animals, though behavioural abnormalities including a reduced exploratory behaviour were observed. Moreover, the mouse mutant model Quaking has been reported as a spontaneous *Park2* knockout, which additionally lacks the PRKN2 co-regulated gene *Pacrg*[68]. Homozygote *quaking* mutants are characterised by dysmyelination in the CNS, resulting in phenotypes including movement disorders, tremors and seizures [68].

In *Drosophila*, the loss of *park* (homologue of *PARK2*) results in reduced cell size and number, infertility, reduced lifespan, movement and flying disorders, and increased sensitivity to oxidative stress [69]. However, even as adults, these *park* mutant flies do not display age-dependent neuron loss in the brain.

Collectively, the mouse and *Drosophila* PRKN2 models will serve as an invaluable tool for understanding the biological role of PRKN2 and may provide important clues to the molecular mechanisms of Parkinson's disease.

## UDPs as drug targets

Inhibitors of the 26S proteasome, such as bortezomib, are successfully being used in the treatment of certain cancers [70]. However, drug targeting of UDPs certainly has several potential advantages over the use of proteasome inhibitors since such drugs would likely be more specific.

Devising efficient inhibitors for non-enzymatic proteins such as the UBL/UBA proteins is more difficult than for proteins with enzyme activity, which can be assayed in high-throughput screens. Nonetheless, recent chemical genetic screens in *Xenopus* extracts performed by Verma *et al.* at Caltech have identified a group of relatively small molecules, called ubistatins, which inhibit the turnover of cyclin-B and the cdk-inhibitor Sic1 *in vitro* by inhibiting the binding of ubiquitylated proteins to substrate shuttles [71]. Accordingly, biochemical assays revealed that the ubiquitin-independent degradation of the proteasome substrate ornithine decarboxylase is not affected by ubistatins indicating that ubistatins may provide a more specific alternative to proteasome inhibitors in cancer treatment. A much more detailed knowledge of the specificity of UBL/UBA proteins and other proteasome co-factors will be necessary before development of drugs targeting UBL/UBA proteins can occur and thus alleviate the neurological disorders associated with these proteins.

As for many loss-of-function mutations in disease-connected E3 enzymes, it is difficult to envision small molecules that could reactivate the mutant PRKN2 and thereby promote neuronal survival in patients suffering from AR-JP. However, a gene therapeutic approach to replace the dysfunctional *PARK2* gene with a wild-type copy could perhaps provide a cure in the future.

In the case of von Hippel-Lindau disease, small molecule activators of mutant VHL may be able to sustain HIFα degradation and prevent tumour angiogenesis. However, it was found that the molecular chaperone Hsp90 protects certain VHL substrates from proteasomal degradation [72] and cycloheximide decay assays revealed that HIFα is degraded by a VHL-independent mechanism in the presence of Hsp90 inhibitors [73]. Hsp90 inhibitors, such as benzoquinone ansamycin 17-allylamino-17-desmethoxygeldanamycin (17-AAG), could therefore be exploited in the treatment of patients with renal carcinomas, an idea currently being assessed in multi-institutional phase I trials [73]. For a review on Hsp90 inhibitors in drug development, please refer to Maloney and Workman [74].

## New frontiers in drug discovery

It is evident that the UBL/UBA proteins and PRKN2 appear to be directly involved in the etiology of cancer and neurodegenerative diseases. Although this is by no means a general feature of the UDPs, it highlights the importance of the ubiquitin system in maintaining an appropriate intracellular protein milieu.

With respect to the UBL/UBA proteins, one major issue that remains unresolved relates to their specificity. Though some details of their substrate selectivity have emerged [17], a more comprehensive picture is needed in order to fully validate them as drug targets. The same is the case for many of the other UDPs encoded in the human genome [11], some of which may also be involved in human disease.

In relation to PRKN2, identifying novel substrates of its E3 activity and resolving its proteasome-dependent and/or -independent functions (especially relating to endocytosis, trafficking and signalling via Eps15) may shed some light on the molecular mechanism of Parkinson's disease and hopefully lead to novel therapeutic approaches.

## Abbreviations

Aβ, amyloid β-protein; AR-JP, autosomal recessive juvenile parkinsonism; CBC, Cullin–Elongin-BC; DUB, deubiquitylating enzyme; EGF, epidermal growth factor; EGFR, epidermal growth factor receptor; GABA_A_, γ-aminobutyric acid A; HIFα, hypoxia-inducible transcription factor α; SCA1, spinocerebellar ataxia type 1; UBA, ubiquitin-associated domain; UBL, ubiquitin-like; UDP, UBL domain protein; UIM, ubiquitin-interacting motif; VHL, von Hippel-Lindau.

## Competing interests

The authors declare that they have no competing interests.

## Publication history

Republished from Current BioData's Targeted Proteins database (TPdb; ).    
